# Manual kidney stone size measurements in computed tomography are most accurate using multiplanar image reformatations and bone window settings

**DOI:** 10.1038/s41598-021-95962-z

**Published:** 2021-08-12

**Authors:** Robert Peter Reimer, Konstantin Klein, Miriam Rinneburger, David Zopfs, Simon Lennartz, Johannes Salem, Axel Heidenreich, David Maintz, Stefan Haneder, Nils Große Hokamp

**Affiliations:** 1 Department of Diagnostic and Interventional Radiology, Faculty of Medicine and University Hospital Cologne, University of Cologne, Kerpener Str. 62, 50937 Cologne, Germany; 2 Department of Radiology, Harvard Medical School, Massachusetts General Hospital, 55 Fruit St, White 270, Boston, MA 02114 USA; 3 Department of Urology, Faculty of Medicine and University Hospital Cologne, University of Cologne, Kerpener Str. 62, 50937 Cologne, Germany

**Keywords:** Ureter, Renal calculi, Three-dimensional imaging

## Abstract

Computed tomography in suspected urolithiasis provides information about the presence, location and size of stones. Particularly stone size is a key parameter in treatment decision; however, data on impact of reformatation and measurement strategies is sparse. This study aimed to investigate the influence of different image reformatations, slice thicknesses and window settings on stone size measurements. Reference stone sizes of 47 kidney stones representative for clinically encountered compositions were measured manually using a digital caliper (Man-M). Afterwards stones were placed in a 3D-printed, semi-anthropomorphic phantom, and scanned using a low dose protocol (CTDI_vol_ 2 mGy). Images were reconstructed using hybrid-iterative and model-based iterative reconstruction algorithms (HIR, MBIR) with different slice thicknesses. Two independent readers measured largest stone diameter on axial (2 mm and 5 mm) and multiplanar reformatations (based upon 0.67 mm reconstructions) using different window settings (soft-tissue and bone). Statistics were conducted using ANOVA ± correction for multiple comparisons. Overall stone size in CT was underestimated compared to Man-M (8.8 ± 2.9 vs. 7.7 ± 2.7 mm, *p* < 0.05), yet closely correlated (*r* = 0.70). Reconstruction algorithm and slice thickness did not significantly impact measurements (*p* > 0.05), while image reformatations and window settings did (*p* < 0.05). CT measurements using multiplanar reformatation with a bone window setting showed closest agreement with Man-M (8.7 ± 3.1 vs. 8.8 ± 2.9 mm, *p* < 0.05, *r* = 0.83). Manual CT-based stone size measurements are most accurate using multiplanar image reformatation with a bone window setting, while measurements on axial planes with different slice thicknesses underestimate true stone size. Therefore, this procedure is recommended when impacting treatment decision.

## Introduction

Urolithiasis is highly prevalent in developed countries and has a relevant impact on quality of life. It occurs in up to 15% of the population, showing an increment in incidence and prevalence over the last decades with a risk of recurrence as high as 50%^1^. In current guidelines, non-contrast computed tomography (CT) is recommended as the modality of choice for the diagnosis of urolithiasis, preferably conducted in low-dose technique. CT has a high sensitivity and specificity regarding detection of stones and provides information on stone localization and size which influence treatment decision between conservative, pharmacological and invasive options^2,3^.

Technical advances in CT imaging include the implementation of new iterative reconstruction algorithms into clinical routine^4^. As a consequence, filtered back-projection (FBP) has gradually been replaced by hybrid- and model-based iterative reconstruction algorithms (HIR and MBIR, respectively), which enable a reduction of radiation dose while maintaining or improving image quality and diagnostic accuracy^4–7^. So far, a few studies demonstrated size and volume measurements of kidney stones to be unaffected by radiation dose, comparing normal-dose and low-dose protocols as low as 2 mGy^8,9^.

Pertaining to image reconstruction methods, a recent study showed closest agreement between CT-based and real kidney stone size when employing a model-based iterative reconstruction algorithm and a sharp image kernel, while radiation dose and denoising levels did not have a significant influence on size measurements^10^. However, even if reconstruction parameters are defined, different slice thicknesses and/or standard image orientations (axial, coronal, sagittal) as well as different window settings (bone versus soft tissue) may impact stone size measurement. This is of particular importance as current guidelines emphasize the role of size assessment for treatment decision by means of the longest diameter. Opposed to these recommendations, no guidance is provided and measurement strategies in routine operations are predominantly driven by individual preference^11–13^. Only recently, it has been shown that kidney stone volume might be a better predictor of treatment outcome, which is known to be time-consuming and hence inapplicable for clinical routine^2,7,14,15^. Another approach to take the irregular 3-dimensional structure of kidney stones into account are multiplanar reformatations (MPR), which enable views in any spatial orientation needed, e.g. alongside the longest axis of the kidney stone^16,17^.

In this study, we aimed to comprehensively evaluate the influence of different reconstruction algorithms (HIR, MBIR), image reformatations (axial, MPR), slice thicknesses (MPR, 2 mm, 5 mm) and window settings (soft-tissue, bone) on accuracy of kidney stone size measurements.

## Methods

47 kidney stones were included in this retrospective study, which was classified as non-human research by the local institutional review board (Ethikkomission der Medizinischen Fakultät der Universität zu Köln). The kidney stones were obtained from the local laboratory at the university hospital of cologne, who collected them over the last years. All methods were performed in accordance with the relevant guidelines and regulations. The reference standard of stone size was determined by manually measuring the long-axis diameter using a digital caliper (Man-M) and only stones with a long axis diameter > 3 mm were included. Stone compositions as determined by infrared spectroscopy comprised the clinically encountered spectrum: brushite (n = 6), cysteine (n = 6), dahllite (n = 2), struvite (n = 4), uric acid (n = 10), weddellite (n = 7), whewellite (n = 7) and xanthine stones (n = 5), (Table 1). These kidney stones have previously been included in another study^10^.

**Table 1:** Manual measurements and CT-based measurements of the longest diameter regarding different kidney stone composition indicated as mean ± standard deviation (range).

Stone composition	N	Man-M (mm)	Overall CT-based (mm)
Calcific	22	8.3 ± 2.7 (4.0–13.3)	7.6 ± 2.3 (4.0–14.2)
Cysteine	6	8.0 ± 1.8 (6.3–11.0)	7.4 ± 2.2 (3.8–12.0)
Struvite	4	10.8 ± 3.2 (6.3–15.0)	10.3 ± 3.5 (4.1–18.0)
Uric acid	10	9.2 ± 3.6 (4.9–13.5)	7.1 ± 3.0 (2.7–16.2)
Xanthine	5	9.5 ± 2.1 (6.6–12.8)	7.8 ± 2.4 (5.1–14.6)

*N* number, *Man-M* manual measurements.

These kidney stones have previously been included in another study (10).

### Phantom design

**Figure 1:**
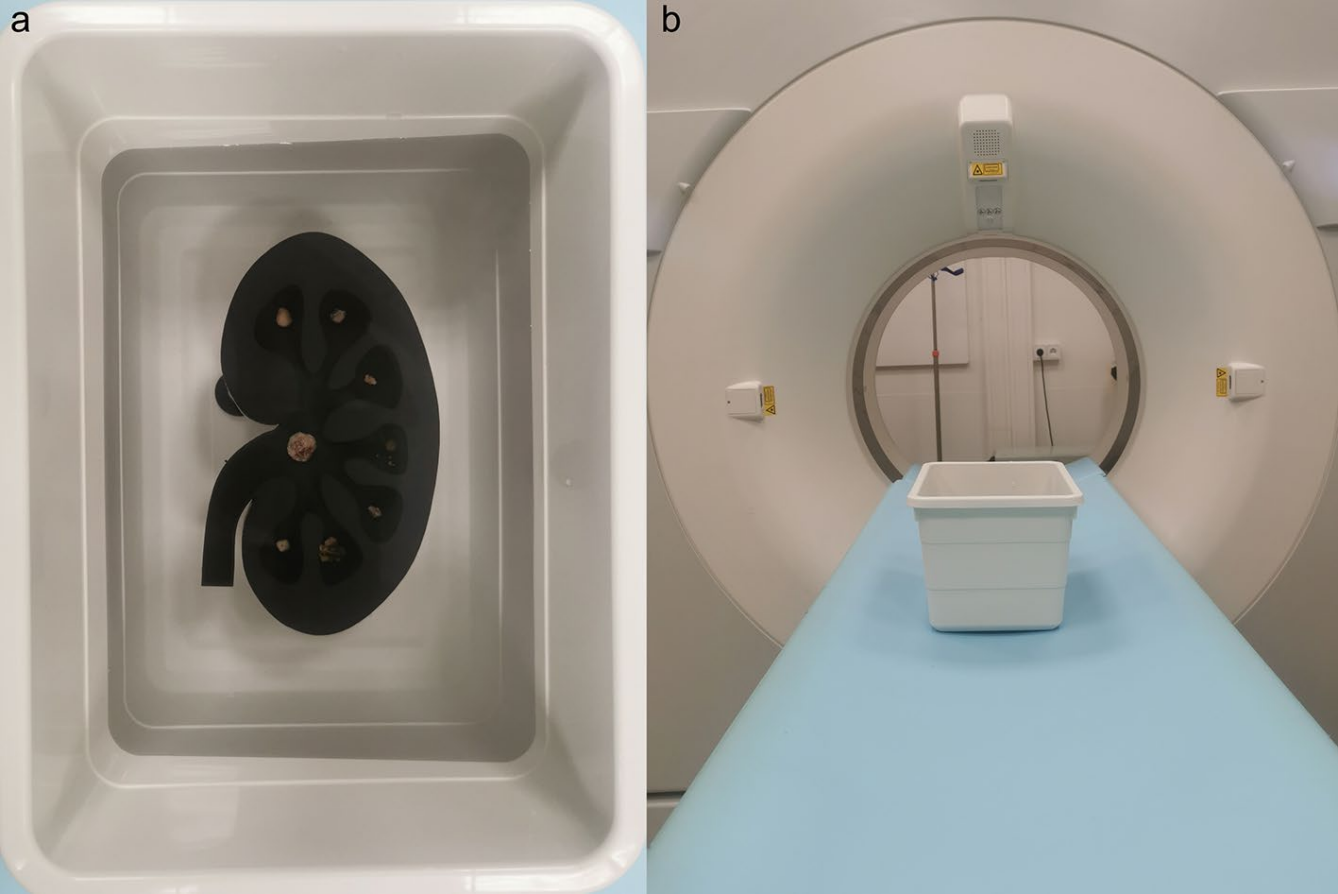
Semi-anthropomorphic phantom with kidney stones placed in a plastic box filled with water. Subsequently, CT scans were performed using a CTDI_vol_ of 2 mGy (**A**,**B**).

All stones were scanned in an ex-vivo setup consisting of a semi-anthropomorphic phantom filled with a layer of gelatin (Oetker, Bielefeld, Germany) and a plastic box filled with water (dimensions: 15 × 18 × 24.5 cm). The phantom with the shape of a kidney was 3D-printed using a stereolithography printer with standard resin (Form 2, FormLabs, Somerville, USA) following a design, which was created using a standard CAD-Software. Attenuation from the resin reached approximately 120 HU. A maximum of 8 stones per scan were placed on the surface of a 5 mm thick gelatin layer, ensuring accurate stone size assessment due to its water-equivalent Hounsfield units (HU) and a sufficient distance to the phantom’s base-plate. Subsequently, the phantom was placed in the plastic box filled with water and the CT scans were performed (Fig. 1). This ex-vivo setup has previously been included in another study^10^.

### Scanning parameters and image reconstruction

All scans were performed on a 64-row spectral computed tomography scanner (IQon; Philips Healthcare, Best, The Netherlands). Scan parameters were as follows: tube current time product 41 mAs, tube voltage 120 kVp, volumetric computed tomography dose index (CTDI_vol_) 2 mGy, pitch 0.80, rotation time 0.75 s and collimation 64 × 0.625 mm. This imaging data have previously been included in another study^10^. Images were reconstructed using a hybrid-iterative reconstruction algorithm (HIR, iDose^4^; Philips Healthcare) and a model-based iterative reconstruction algorithm (MBIR, IMR; Philips Healthcare) with a sharp image kernel, medium denoising level (kernel B and Sharp, denoising level 4/7 and 2/3 for HIR and MBIR, respectively) and different slice thicknesses (0.67 mm, 2 mm and 5 mm) with identical section increments, respectively.

### Kidney stone measurements

Size measurements were independently performed by 2 licensed radiologists using a clinical DICOM-Viewer (Impax EE R20; Agfa Healthcare). The maximum diameter was measured on clinically established 2 mm and 5 mm axial image reformatations and on multiplanar reformatations (MPR) of thin slices (0.67 mm) using a soft-tissue window setting (width = 360 HU, level = 60 HU) and a bone window setting (width = 1720 HU, level = 530 HU), respectively. For measurements using MPR, the readers were asked to choose the image plane alongside the largest diameter of the stones taking the irregular 3-dimensional structure into account.

### Statistical assessment

All analyses were carried out using JMP Software (V14 SAS Institute, Cary, NC, USA) unless differently specified below. To allow for comparison between different reconstruction algorithms, image reformatations, slice thicknesses and window settings, ANOVA was used and adjusted for multiple comparisons if appropriate. Correlation between reference standard and CT measurements of the longest diameter was determined with Pearson’s correlation. Inter-rater reliability was determined by means of intra-class correlation estimates (ICC) using R Studio (Version 1.1.456; http://rstudio.org/download/desktop) based on a mean of 2 raters, consistency, 2-way mixed-effects model^18^. Inter-rater agreement was evaluated as described earlier: excellent (ICC > 0.8), good (ICC > 0.6), moderate (ICC > 0.4), and poor agreement (ICC < 0.4)^19^. A *p* value < 0.05 was considered significant. Results are presented as mean ± standard deviation.

## Results

Overall, the intraclass correlation between the 2 independent readers was 0.985 with a 95% confidence interval of 0.982–0.987, indicating an excellent inter-reader reliability. ICC varied from 0.985 to 0.994 for the different approaches/reconstructions.

### Kidney stone measurements

Stone size as determined using a digital caliper served as reference standard with an average stone size of 8.8 ± 2.9 mm ranging from 4 to 15 mm, while CT-based measurements systematically underestimated stone size (7.7 ± 2.7 mm; when averaging all measurements; *p* < 0.05); yet, Man-M and CT-based measurements showed a good correlation (*p* < 0.05, *r* = 0.70), (Table 2).

**Table 2:** Kidney stone size measurements regarding different reconstruction algorithms, image reformatations, slice thicknesses and window settings indicated as mean ± standard deviation (range) [Pearson correlation coefficient].

Reconstruction algorithm	Image reformatation	Slice thickness	Window setting	CT-based stone size measurements
HIR	Axial	2	Soft-tissue	7.5 ± 2.2 (3.1–12.9) [0.66]
			Bone	6.9 ± 2.2 (2.9–12.5) [0.64]
		5	Soft-tissue	7.1 ± 2.4 (2.8–13.5) [0.69]
			Bone	6.7 ± 2.3 (2.7–13.6) [0.70]
	MPR	0.67	Soft-tissue	9.2 ± 3.0 (4.7–17.8) [0.84]
			Bone	8.6 ± 3.1 (4.3–18.1) [0.83]
MBIR	Axial	2	Soft-tissue	7.5 ± 2.2 (3.3–12.9) [0.71]
			Bone	7.1 ± 2.2 (3.1–13.0) [0.72]
		5	Soft-tissue	7.1 ± 2.3 (3.1–13.7) [0.70]
			Bone	6.9 ± 2.2 (3.0–13.6) [0.71]
	MPR	0.67	Soft-tissue	9.3 ± 3.0 (4.9–17.9) [0.84]
			Bone	8.8 ± 3.1 (4.3–17.6) [0.83]
As compared to manual measurements serving as a reference standard				8.8 ± 2.9 (4.0–15.0)

*HIR* hybrid-iterative reconstruction algorithm, *MBIR* model-based iterative reconstruction algorithm, *MPR* multiplanar reformatation.

#### Reconstruction algorithms

CT-based measurements of stone size did not significantly differ between images reconstructed with HIR and MBIR (7.7 ± 2.7 mm vs. 7.8 ± 2.7 mm, *p* > 0.05). Further, each imaging protocol correlated closely with Man-M (*r* = 0.68 and *r* = 0.70, *p* < 0.05) (Table 2). Largest underestimation of size was up to 6 mm using HIR and MBIR, respectively (Man-M, 13.0 mm; HIR, 7.0 mm; MBIR, 7.0 mm).

#### Image reformatations and slice thicknesses

Measurements using MPR yielded significantly larger stone sizes compared to axial reformatations with a slice thickness of 2 mm and 5 mm, respectively (8.9 ± 3.1 mm vs. 7.2 ± 2.2 mm and 7.0 ± 2.3 mm, *p* < 0.05). In line, stone size measurements using MPR showed a better correlation with Man-M than those performed on 2 mm/5 mm axial images (*r* = 0.83 vs. *r* = 68/*r* = 0.70). On the contrary, CT-based measurements on axial reformatations did not significantly differ between images with a slice thickness of 2 mm and 5 mm (*p* > 0.05) (Table 2). Largest underestimation of size was the same in 2 and 5 mm axial images (6.0 mm), while the same stone was overestimated by up to 3.2 mm using MPR (Man-M, 13.0 mm; axial 5 mm, 7.0 mm; axial, 2 mm, 7.0 mm; MPR, 16.2 mm).

#### Window settings

**Figure 2:**
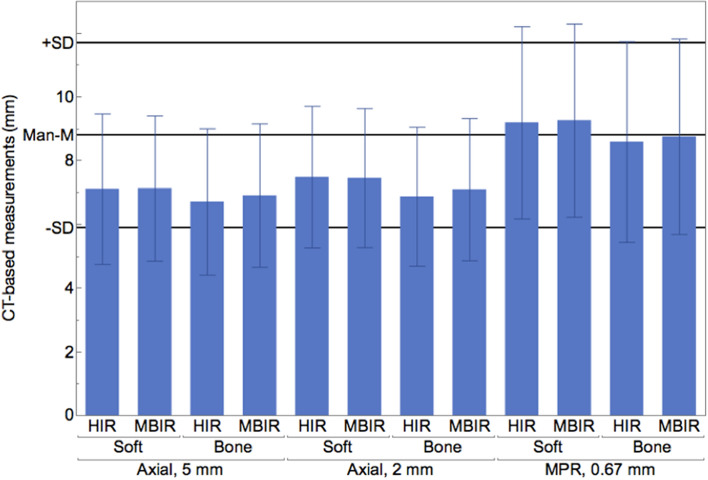
Computed tomography (CT) based measurements of kidney stone size performed on multiplanar reformatations (MPR) using a bone window setting in images reconstructed with a model-based iterative reconstruction algorithm (MBIR) (8.8 ± 3.1 mm) showed closest agreement with Man-M (8.8 ± 2.9 mm) compared to axial reformatations, a soft-tissue window setting and hybrid-iterative reconstruction algorithm.

**Figure 3:**
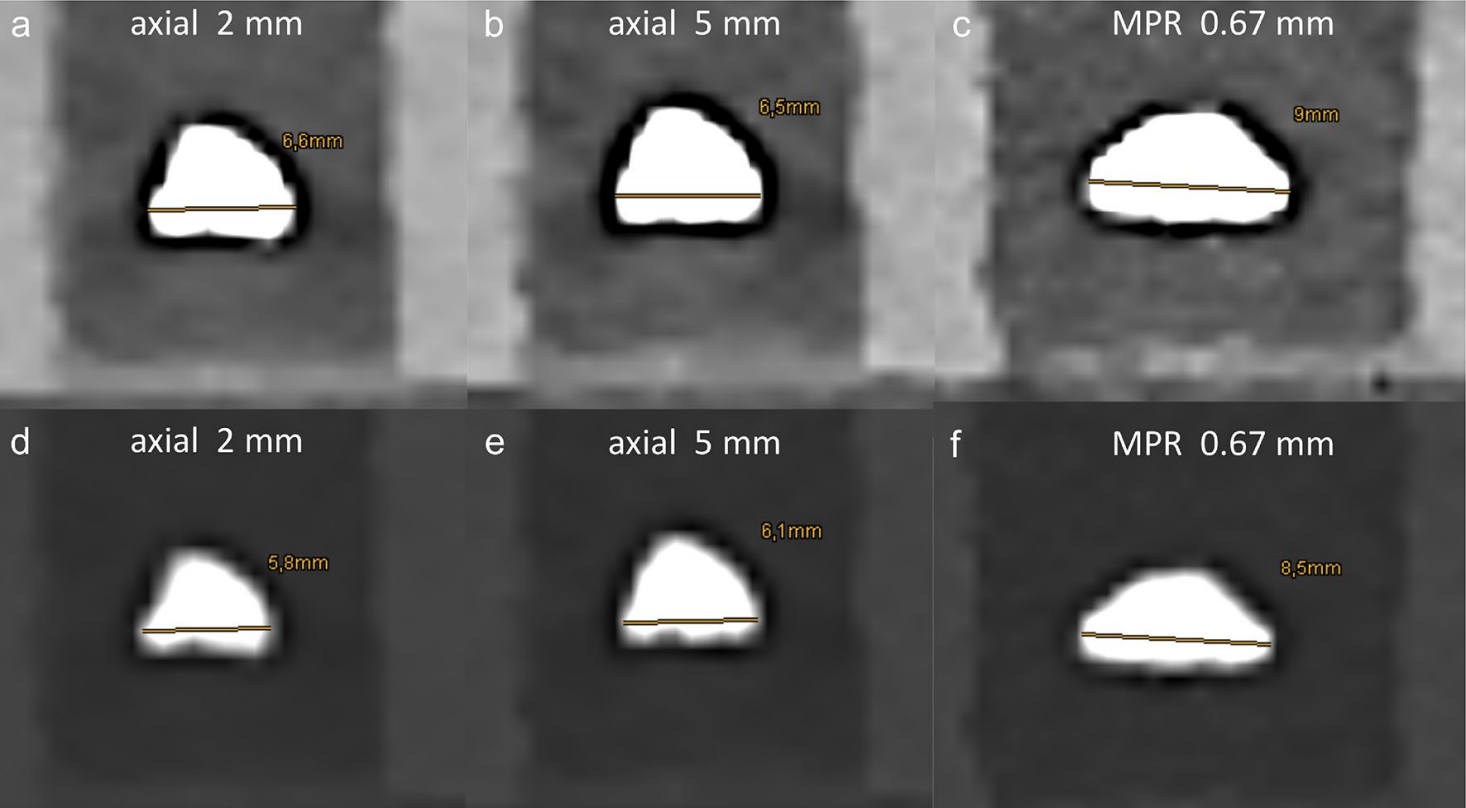
Computed tomography images reconstructed with a model-based iterative reconstruction algorithm of one kidney stone with a manually measured longest diameter of 8.61 mm, illustrating the influence of different image reformatation, slice thickness and window setting on size measurements (**A**–**F**). Irrespective of slice thickness, size measurements using axial reformatations underestimated true stone size (**A**,**B**,**E**,**F**), while measurements were higher using a soft-tissue window (**A**–**C**) vs. a bone window setting (**D**–**F**). Most accurate measurements were obtained using multiplanar reformatation with a bone window (**F**).

Stone size measurements using bone window settings were significantly lower as compared to soft-tissue window settings (7.5 ± 2.7 mm vs. 7.9 ± 2.7 mm; when averaging all other parameters, *p* < 0.05). Regarding MPR, measurements using bone window settings showed a closer agreement with Man-M, while measurements using soft-tissue window settings slightly overestimated stone size without reaching a significant difference (*p* > 0.05; e.g. Man-M vs. MBIR (bone window) vs. MBIR (soft-tissue window): 8.8 ± 2.9 mm vs. 8.8 ± 3.1 vs. 9.3 ± 3.0 mm), (Figs. 2, 3; Table 2). Largest underestimation of size was up to 6 mm using bone and soft-tissue window settings, respectively.

## Discussion

This study investigated the influence of different reconstruction algorithms, image reformatations, slice thicknesses and window settings on measurement accuracy of kidney stone size. We found that stone size as determined in CT differed between image reformatations and window settings. Measurements using axial reformatations underestimated true stone size irrespective of slice thickness. Best agreement with true stone size was yielded using multiplanar reformatation with a bone window setting in images reconstructed with a model-based iterative reconstruction algorithm.

Recommendations on how to perform kidney stone size measurements in CT are rare and missing in current guidelines^2,20^. This is one of the reasons for the substantial heterogeneity found in the literature in this regard. Determination of most reliable measurement techniques and standardization of these procedures are key for obtaining precise and comparable results throughout different studies and in clinical routine^7,20–22^. While in recent years there has been rapid development towards reducing radiation dose using various image reconstruction techniques, the influence of these more refined techniques on kidney stone size measurements are largely elusive^5–7,10^.

So far, few studies compared kidney stone size measurements using different reconstruction algorithms without providing a reference standard. They reported no differences in stone size and volume between FBP and HIR^23,24^, while lower measurements were found using MBIR as compared to FBP^9^.

As expected, stone size measurements using MPR were more accurate as compared to axial reformatations, of which the latter underestimated true stone size. This is due to the possibility to assess stone size of the irregular 3-dimensional kidney stones in any spatial orientation, e.g. alongside their longest diameter as performed in this study. The irregular 3-dimensional structure of the kidney stones hampers the use of defined reformatations, yet the assessment of the largest diameter on axial and/or coronal planes is clinical routine^7,15,21^. However, varying results have been reported regarding the clinical benefit of these standard reformatations for size measurements. Kadihasanoglu et al. reported an association of the coronal stone diameter with stone passage vs. need for invasive treatment, while Bandi et al. an assoziation between the axial stone diameter and clinical outcome after extracorporeal shock wave lithotripsy^11,15^. Other studies showed that kidney stone volume might be a better predictor of treatment outcome and therefore an even better criterion for treatment decision, whereas current guidelines only recommend to determine the longest diameter^2,7,14,15^; possibly, as volumetric assessment appears unlikely during routine operations.

On the other hand, it appears surprising that slice thickness of axial reformatations did not significantly impact size measurements in our dataset, since it was previously reported that smaller slice thicknesses resulted in more accurate and less variable stone size measurements as well as more accurate volume measurements^12,22,25^. Last but not least, our results are in line with few earlier studies showing more accurate and less variable results using a bone window setting over a soft-tissue window setting^12,13^.

Besides the importance of low-dose, non-contrast CT as the imaging modality of choice in suspected urolithiasis; a more recent innovation, dual-energy CT (DECT) demonstrated its beneficial value in the imaging of urolithiasis enabling an increased material separation. DECT allows for reconstruction of virtual monoenergetic images which are known to reduce blooming of calcified structures and may impact size measurements^26^. Furthermore, DECT provides additional information about kidney stone composition, which may be exploited with regards to treatment decision making^7,27^. DECT also allows for the reconstruction of virtual non-contrast (VNC) images by virtually removing the contrast media from contrast-enhanced images. These reconstructions hold the potential to facilitate the differentiation between urolithiasis and phlebolites in the pelvis adjacent to the ureter or urinary bladder by virtually removing the contrast media in an excretory phase^28,29^.

Aside from the retrospective study design, some limitations of this study need to be discussed. First, we only included a limited number of kidney stones which became necessary to the required amount of conducted measurements; yet, the sample size is comparable to earlier investigations and considered to be sufficient with regards to stone composition, shape and size^1,12,13,22^. Second, we adapted radiation dose from previous in-vivo and ex-vivo studies as well as from our institutional low-dose protocol for unenhanced urolithiasis CT^10,27,30^. However, particularly in the low dose setting, image quality obtained in our ex vivo set-up does not necessarily translate into in-vivo applications due to a comparably small sized phantom and perfect homogeneous attenuation characteristics. Hence and third, our ex-vivo findings need to be validated in-vivo before implementation in clinical routine and guidelines is possible. The ex-vivo design does not account for preferential growth directions possibly encountered in-vivo (i.e., preference towards maximum dimension along the ureter and hence in coronary plane). On the other hand, this set-up allowed us to provide true reference measurements which is considered a particular strength of our study. Unlike earlier studies considering the irregular 3-dimensional structure of kidney stones by employing volumetric assessments, we investigated the longest diameter using standard (and available) reconstructions as well as MPR as these more likely represent clinical routine. Last, we compared different reconstruction techniques using a single scanner, whereas an inter-vendor comparison and an association with clinical outcome were out of scope of this study.

## Conclusions

CT measurements using axial reformatations tend to systematically underestimate size measurements of kidney stones. While image reformatation and window settings affect measurement accuracy, hybrid and model-based reconstruction algorithms and different slice thicknesses using axial reformatations demonstrate no influence on stone size measurements. We found closest agreement between CT-based measurements and true stone size using multiplanar reformatations with a bone window setting in images reconstructed with a model-based iterative reconstruction algorithm. Therefore, these settings should undergo systematic evaluation in-vivo and are recommended for studies reporting stone size measurements based on CT.

## Data Availability

The data that support the findings of this study are available from the corresponding author upon reasonable request.

## Abbreviations

CT: Computed tomography
DECT: Dual-energy computed tomography
FBP: Filtered back-projection
HIR: Hybrid-iterative reconstruction algorithm
ICC: Intra-class correlation estimates
Man-M: Manual measurements
MBIR: Model-based reconstruction algorithm
MPR: Multiplanar reformatation
